# Targeted Therapy in Acute Myeloid Leukemia: Current Approaches and Novel Directions

**DOI:** 10.3390/jpm16030169

**Published:** 2026-03-20

**Authors:** Kaitlyn H. Ko, Rebecca Gelfer, Justin C. Wheat, Sheng F. Cai

**Affiliations:** 1 Human Oncology and Pathogenesis Program, Memorial Sloan Kettering Cancer Center, New York, NY 10065, USA; 2 Department of Medicine, Leukemia Service, Memorial Sloan Kettering Cancer Center, New York, NY 10065, USA

**Keywords:** acute myeloid leukemia, MENIN, IDH, BCL2, FLT3, precision oncology, venetoclax, clonal evolution, molecular profiling

## Abstract

Acute myeloid leukemia (AML) is a molecularly heterogeneous neoplasm of hematopoietic stem and progenitor cells. The advent of high-resolution genomic sequencing has uncovered several genetic drivers of AML which spurred a surge of therapies that target the disease at a mutational, clonal, or epigenetic level. Currently, the molecular profiling of AML patients before treatment is commonplace and crucial for ensuring that patients receive the most optimal therapy for any driver mutations they may have. Here, we detail the current targeted therapies available for AML: specifically, those targeting the BCL2 family (venetoclax), FLT3 (midostaurin, gilteritinib, quizartinib), IDH1/2 (enasidenib, ivosidenib), and MENIN (revumenib, ziftomenib). In addition, we outline potential mechanisms of resistance against these therapies, as well as efforts being taken to prevent or bypass them.

## 1. Introduction

Acute myeloid leukemia (AML) is an aggressive hematologic malignancy characterized by poor long-term outcomes. With the advent of genomic sequencing, the key oncogenic drivers of AML have now been well characterized, revealing actionable dependencies [1]. While conventional chemotherapy has made a significant contribution to improving AML outcomes, many patients either cannot tolerate intensive chemotherapy or fail to respond after multiple cycles. Therapeutics targeting known genetic drivers have made rapid advances in patient outcomes within the last ten years. The availability of routine targeted sequencing now refines diagnosis and risk stratification and guides therapy selection, enabling a more personalized, effective approach to care.

This review highlights four key areas of targeted therapy in acute myeloid leukemia: BCL2 family-, FLT3-, IDH-, and MENIN-directed agents. We assess the key studies that reveal their efficacy and, in some cases, lead to FDA approval. We also review next generation therapeutics and common resistance mechanisms to comprehensively assess the landscape of targeted agents.

## 2. BCL2 Family Member-Targeting Agents

Healthy cells that experience stress are able to undergo apoptotic cell death. However, many AML cells can evade cell death through dysregulation of apoptotic family members. There are two main pathways for apoptosis: the extrinsic pathway and the intrinsic pathway. In the extrinsic pathway, cell death is initiated by binding of extracellular ligands to FS-7-Associated-Surface Antigen (FAS), TRAIL, or TNFα receptors, leading to recruitment of the adapter protein FAS-associated death domain (FADD) and subsequent activation of caspase-8. This cascade then activates caspases 3 and 7 which triggers apoptosis [2,3]. In the intrinsic pathway, cell death is regulated by BCL-2 family members and is triggered by cell autonomous factors (such as DNA damage, unfolded proteins, and oxidative damage). In response to apoptotic stimuli, BH3-only proteins are upregulated. Activator BH3-only proteins (e.g., BIM, BID) directly activate pro-apoptotic effectors BAX and BAK, while sensitizer BH3-only proteins neutralize pro-survival proteins (BCL-2, BCL-XL, MCL-1), enabling BAX and BAK activation. This leads to mitochondrial outer membrane permeabilization (MOMP), release of cytochrome c, and subsequent caspase-mediated cell death [3]. Early studies revealed that AML cells upregulate BCL-2 and upregulation of MCL-1 and BCL-XL confers chemotherapy resistance [4,5]. Furthermore, certain malignant cells were found to be more ready for apoptosis (“primed for apoptosis”) than normal hematopoietic cells, which supports a mitochondrial basis for differences in chemotherapeutic sensitivity [6]. Thus, there was strong interest in targeting this pathway to induce apoptosis in AML cells.

### 2.1. Venetoclax

Venetoclax is a first-in-class BH3-mimetic FDA-approved for hematologic malignancies that binds BCL-2 with sub-nanomolar affinity [7]. Venetoclax competes with pro-apoptotic BH3 motifs for BCL-2 binding, releasing the pro-apoptotic protein BIM which then activates BAX and BAK, triggering a cell death cascade and promoting apoptosis in cells primed for death. In 2016, venetoclax received FDA approval for treatment of patients with chronic lymphocytic leukemia (CLL) with 17p deletion who received prior therapy following robust clinical trial data, revealing an overall response rate of 79% [8]. In preclinical studies, venetoclax induced rapid cell death in AML cell lines and primary patient samples [9]. The strong preclinical AML data and the FDA approval in CLL prompted clinical evaluation of venetoclax as an AML therapeutic. An initial study performed in a cohort of relapsed/refractory AML patients demonstrated only modest outcomes with venetoclax monotherapy, with an overall response rate of 19% and median duration of complete response (CR) of 48 days [10]. However, subsequent preclinical data revealed strong synergy of venetoclax with the hypomethylating agent (HMA) azacytidine [11]. This observation led to a clinical trial (NCT02203773) investigating venetoclax in combination with azacitidine or decitabine in newly diagnosed elderly patients with AML who were deemed unfit for intensive chemotherapy. Treatment with the HMA–venetoclax combination resulted in a 68% overall response rate (ORR), with 37% of patients achieving CR, a stark improvement compared historically to previous standard-of-care regimens [12]. This trial, along with strong results from another trial (NCT02287233) evaluating venetoclax in combination with low-dose cytarabine, led to FDA approval of venetoclax in combination with azacitidine, decitabine or low-dose cytarabine (LDAC) for the treatment of newly-diagnosed AML in adults 75 years or older, or who have comorbidities that preclude use of intensive induction chemotherapy. The follow-up VIALE-A phase III trial confirmed the outcomes for venetoclax plus azacitidine with the combination resulting in an ORR of 66.4% compared to 28.3% in the azacitidine-only group (*p* < 0.001). The median overall survival (OS) was 14.7 months in the azacitidine–venetoclax arm and 9.6 months in the control arm (*p* < 0.001) [13]. Importantly, composite CR rates were improved across all AML genomic risk groups, including patients with adverse cytogenetic risk, secondary AML, and high-risk molecular mutations, all of which have historically shown lower response rates to conventional chemotherapy. Following these findings, a number of trials were conducted to assess the role of venetoclax in combination with gilteritinib (FLT3 inhibitor) [14], ivosidenib (IDH1 inhibitor) [15,16], and as first-line treatment in combination with standard induction chemotherapy in fit AML patients [17]. These trials revealed improvements in disease-related outcomes and support further investigation to limit potential drug toxicities associated with combinatorial therapy. Taken together, there are strong preclinical and clinical trial data supporting the clinical utility of HMA–venetoclax combinations in AML.

Despite the excitement surrounding venetoclax-based combinations, a substantial subset of patients exhibit primary refractoriness or relapse, and defining mechanisms of resistance remains an active area of investigation. In addition to clonal evolution driving disease progression and therapeutic resistance, another well-established theme is a shift in anti-apoptotic priming away from BCL-2, most commonly through increased reliance on MCL-1, which can blunt venetoclax-induced apoptosis [10]. However, despite promising preclinical evidence [18], clinical trials utilizing MCL-1 inhibitors demonstrated high rates of on-target cardiotoxicity, constraining effective dosing [19]. Other described mechanisms of resistance include rewired fatty acid metabolism [20], oncogenic Ras signaling [21], and alterations in mitochondrial reprogramming and oxidative metabolism [22].

### 2.2. Second-Generation BCL-2 Inhibitors

Several second-generation BCL-2 inhibitors with increased potency are currently under investigation in myeloid malignancies. While venetoclax is the only currently FDA-approved BCL-2 inhibitor, lisaftoclax (APG-2575) has advanced farthest in myeloid malignancies, with promising preclinical data and phase Ib/II studies evaluating its combination with azacitidine. Although these clinical trials are ongoing, early data suggests promising results, as the overall response rate in patients with relapsed/refractory AML/mixed phenotype acute leukemia (MPAL) treated with lisaftoclax for 28 days was 38.9%. Intriguingly, in venetoclax-refractory AML patients, the overall response rate was 29.2% [23,24], suggesting potential activity in venetoclax-exposed patients, though cross-resistance mechanisms remain undefined. Additionally, sonrotoclax (BGB-11417) recently received breakthrough therapy designation for relapsed/refractory mantle cell lymphoma based on promising efficacy data and is being evaluated in both frontline and relapsed AML settings, including in combination with azacitidine or intensive chemotherapy [25]. Side effects of second-generation BCL-2 inhibition in combination with azacitidine in these early trials was mainly associated with myelosuppression (neutropenia, thrombocytopenia, anemia) and related infections/febrile neutropenia, with gastrointestinal symptoms and fatigue common as well. Collectively, while these agents show promise, their role in AML remains investigational and will require efficacy and survival data from larger trials.

### 2.3. Navitoclax

Navitoclax (ABT-263) is an orally bioavailable small-molecule inhibitor of the BCL-2 family, but unlike venetoclax, it targets both BCL-2 and BCL-XL. Through its dual antagonist properties, it was thought that its broader target profile may make it more likely to overcome venetoclax-mediated resistance. Early phase I trials have interrogated the role of navitoclax in hematologic malignancies. One ongoing phase I trial investigated triplet combination therapy of venetoclax, navitoclax, and decitabine in relapsed/refractory AML; early data has suggested this combination is tolerated and 20% of patients achieve CR, CRh (complete remission with partial hematologic recovery), or CRi (complete remission with incomplete hematologic recovery), with some patients bridged to allogeneic stem cell transplantation [26]. Despite these data, BCL-XL is known to be required for platelet survival and on-target thrombocytopenia occurs in a substantial number of patients, limiting clinical utility [27,28].

### 2.4. FLT3 Targeting Agents

The FMS-like tyrosine kinase 3 (*FLT3*) gene is one of the most frequently mutated genes in patients with AML [29]. The *FLT3* gene encodes a transmembrane FLT3 receptor tyrosine kinase (RTK) in hematopoietic progenitor cells, which allows for normal maturation and proliferation of the hematopoietic compartment [30]. The RTK is activated when the FLT3 ligand (FL) binds to the extracellular domain, inducing receptor dimerization and autophosphorylation of the intracellular kinase domain [31,32]. This consequently activates downstream signaling cascades such as the rat sarcoma (RAS) signal-transduction cascade [33]. *FLT3* mutations cause constitutive activation of the RTK, resulting in aberrant proliferation of the mutated cells [34].

*FLT3* internal tandem duplications (*FLT3*-ITD) were found to achieve this by disrupting the autoinhibitory function of the juxtamembrane domain, whereas tyrosine kinase domain point mutations (*FLT3*-TKD), most commonly involving the activation loop, stabilize the active kinase conformation and impair autoinhibition [35]. ITD mutations are associated with a high rate of relapse and poor prognosis [29], whereas the prognostic effects of TKD mutations appear more variable and are generally considered less adverse than *FLT3*-ITD [36,37,38].

Aplasia following cytotoxic chemotherapy leads to marked increases in circulating FLT3 ligand (FL), which has been hypothesized to preferentially stimulate residual *FLT3*-mutant (*FLT3m*) AML cells, thereby positively selecting for this population [39]. Repeated cycles of chemotherapy may therefore enrich a more FLT3-dependent leukemic clone with a higher mutant allelic burden [40]. On this basis, it has been proposed that first-generation FLT3 tyrosine kinase inhibitors (TKIs), with broader kinome profiles, may be advantageous in newly diagnosed *FLT3m* AML by suppressing a more polyclonal disease state. In contrast, more selective second-generation FLT3 inhibitors might be better suited to later disease stages, including relapsed or refractory (R/R) AML, where FLT3-addicted clones have emerged following ligand-driven selection [41]. Notably, this ligand-dependent biology has also provided a rationale for extending FLT3 inhibition beyond genomically defined *FLT3m* disease, as exemplified by the ongoing phase 3 QuANTUM-Wild trial (ClinicalTrials.gov ID NCT06578247) evaluating quizartinib in combination with chemotherapy as maintenance therapy in patients with newly diagnosed FLT3-ITD-negative AML. Together, these data suggest that both mutational status and treatment-induced FL dynamics shape FLT3 dependency across disease stages. Three FLT3 inhibitors are currently approved by the U.S. Food and Drug Administration for AML: midostaurin (first generation), gilteritinib (second generation), and quizartinib (second generation).

### 2.5. Midostaurin

The first *FLT3* inhibitor to be FDA-approved for *FLT3m* leukemia was midostaurin, to be used in combination with standard induction chemotherapy. A type I FLT3 inhibitor, midostaurin binds the active conformation of the FLT3 tyrosine kinase domain, rendering it active against both *FLT3*-ITD and *FLT3*-TKD AML. Its putative role as a FLT3 kinase inhibitor was identified via small molecule screens [42]. Owing to potent preclinical activity against *FLT3m* cell lines and encouraging early phase studies, the phase 3 RATIFY trial was conducted. Newly-diagnosed patients aged 18–59 years were randomized to receive either midostaurin (50 mg orally twice daily) or placebo with standard induction and consolidation chemotherapy, followed by maintenance therapy with midostaurin or placebo for 12 months. The trial showed significantly improved median overall survival (OS) in patients receiving chemotherapy with midostaurin, as compared with chemotherapy plus placebo (74.7 vs. 25.6 months respectively, *p* = 0.009), and improved event-free survival (EFS 8.2 vs. 3.0 months respectively, *p* = 0.002) [43].

Despite these results, of the 59% of RATIFY patients who achieved CR with midostaurin, almost half relapsed. Schmalbrock et al. analyzed clonal evolution patterns in *FLT3*-ITD AML from the RATIFY and AMLSG 16-10 trials and found that nearly half of patients treated with midostaurin become *FLT3*-ITD negative at the time of disease progression but acquire new mutations in signaling pathways (e.g., MAPK family members) [44]. Nevertheless, 32% of patients retained the *FLT3*-ITD mutation at time of disease resistance or progression, which suggested that either cells acquire resistance via bypassing FLT3 inhibition or that there is insufficient midostaurin inhibitory activity to completely inhibit all *FLT3*-ITD populations.

### 2.6. Gilteritinib

First-generation FLT3 agents, like midostaurin, exhibited pleiotropic multi-kinase activity rather than selective FLT3 inhibition. As a result, their use led to off-target effects and subsequent toxicities. Gilteritinib is among the second generation of FLT3 inhibitors, which were developed to specifically target FLT3 with increased potency and fewer off-target effects. Gilteritinib is currently the only FDA-approved FLT3 inhibitor for patients with relapsed or refractory (R/R) *FLT3m* AML. Preclinically, gilteritinib was shown to be highly selective at inhibiting *FLT3m* cells [45,46]. The phase 3 ADMIRAL trial compared the effects of gilteritinib (120 mg orally once daily) versus salvage chemotherapy for patients with R/R *FLT3m* AML [47]. The median OS was significantly greater for the gilteritinib treatment group (9.3 vs. 5.6 months, *p* < 0.001), and patients treated with gilteritinib monotherapy showed improved CR rates (34% vs. 15.3%) and EFS (2.8 vs. 0.7 months) compared with salvage chemotherapy. The gilteritinib arm also experienced fewer serious adverse effects than those in the chemotherapy arm, although some common adverse effects included febrile neutropenia and anemia.

Gilteritinib is a type I FLT3 inhibitor which binds to the active conformation of the FLT3 kinase [48]. The median OS for ITD and TKD groups in the gilteritinib arm of ADMIRAL trial were 9.3 and 8.0 months, respectively, suggesting that gilteritinib maintains activity in both mutant populations. Because 88% of patients enrolled in ADMIRAL were FLT3 inhibitor-naïve [47], the efficacy of gilteritinib following prior FLT3 inhibitor exposure requires further investigation.

Currently, gilteritinib has not been approved for use in newly diagnosed *FLT3m* AML patients. The phase 3 LACEWING study compared the effects of gilteritinib plus azacitidine versus azacitidine alone in treating newly diagnosed chemotherapy-ineligible *FLT3m* AML patients [49]. Unfortunately, this study failed to meet the primary endpoint OS at interim analysis. However, preliminary data from the safety cohort of gilteritinib plus azacitidine demonstrated trends towards improvement, showing that 10 of 15 patients achieved composite complete remission (CRc) with a median remission duration of 10.4 months in the ten patients with CRc. Moreover, an ongoing phase 3 trial (HOVON 156 AML, NCT04027309) is evaluating the effects of induction and consolidation treatment combined with gilteritinib versus midostaurin for newly diagnosed *FLT3m* AML patients.

### 2.7. Quizartinib

Quizartinib is a second-generation type II FLT3 inhibitor that was approved by the FDA for treatment of newly diagnosed *FLT3*-ITD AML in July 2023. Preclinically, quizartinib was shown to be potent and highly selective for both *FLT3*-ITD and wild-type cell lines [50,51,52]. The phase 3 QuANTUM-First trial, which led to FDA approval of quizartinib, randomized patients to receive induction and consolidation therapy in combination with quizartinib versus placebo, as well as maintenance monotherapy with either quizartinib or placebo for up to three years [53]. The quizartinib arm had a significantly longer OS than the placebo arm (31.9 months vs. 15.1 months respectively, HR = 0.78, *p* = 0.0324). The CR rates of quizartinib and placebo arms were both 55%, but the median duration of response was much longer for the quizartinib arm than the placebo arm (38.6 months vs. 12.4 months).

## 3. IDH-Targeting Agents

Isocitrate dehydrogenase (IDH) is a metabolic protein commonly mutated in AML [54,55]. Its canonical function is to convert isocitrate to α-ketoglutarate, a key intermediate in the citric acid cycle. Oncogenic gain-of-function mutations most commonly occur at catalytic-site arginine residues (R132 for *IDH1* and R140 or R172 in *IDH2*). These mutations confer a neomorphic function to IDH, converting α-ketoglutarate to oncometabolite 2-hydroxyglutarate (2-HG). 2-HG competitively binds and inhibits α-ketoglutarate-dependent enzymes such as ten-eleven translocation 2 (TET2), leading to DNA hypermethylation, myeloid differentiation blocks, and upon acquiring a secondary driver mutation, leukemic transformation [56]. Based on these studies and the elevated frequency of IDH1/2 mutations in AML [1], there has been significant effort in developing highly potent, mutant-selective IDH1/2 inhibitors with strong clinical benefit.

### 3.1. Enasidenib

Enasidenib (AG-221) is a mutant IDH2-selective inhibitor FDA approved in 2017 for adult patients with R/R *IDH2*-mutant AML. In addition to strong preclinical data [57], approval was based on a single-arm, multicenter clinical trial of enasidenib that included 199 adults with R/R *IDH2*-mutant AML (NCT01915498). After a median follow-up of 6.6 months, the overall response rate was 40.3%, with a median response duration of 5.8 months. Additionally, of the 157 patients who required transfusions at the initiation of the trial, 34% no longer required transfusions during at least one 56-day time period [58]. Despite the significant response, the *IDH2*-mutant clone persisted. The prognostic implication of this remains unclear, but sustained treatment may be required to restrain further clonal proliferation. A notable side effect of IDH inhibitor treatment was differentiation syndrome, a systemic inflammatory condition characterized by fever, dyspnea, pulmonary infiltrates, and pleural/pericardial effusions caused by excessive cytokine release following rapid differentiation of leukemic blasts. Additionally, RAS and receptor tyrosine kinase (RTK) pathway mutations were associated with clinical resistance to IDH inhibition [58]. Following the phase 1/2 trial that led to FDA approval, subsequent clinical trials have further defined the role of enasidenib across disease states and in combination with standard-of-care regimens. The randomized phase 3 IDHENTIFY trial compared enasidenib with conventional care regimens (CCR) in older patients with relapsed/refractory *IDH2*-mutant AML and, although it did not meet its primary overall survival endpoint, demonstrated significant improvements in event-free survival (median, 4.9 enasidenib vs. 2.6 months with CCR, *p* = 0.008) and overall response rate (40.5% vs. 9.9%; *p* < 0.001) [59]. Enasidenib has also shown activity in newly diagnosed older or unfit patients, with a small phase 1/2 study reporting a CR rate of 50% [60]. When evaluating combinatorial therapy, enasidenib with azacitidine in newly diagnosed AML patients performed significantly better than azacitidine alone, with 74% of patients in the enasidenib plus azacitidine combination group and 36% patients in the azacitidine monotherapy group achieving an overall response (*p* = 0.0003) [61]. A small phase 1b/2 trial evaluating enasidenib in combination with venetoclax in relapsed/refractory AML patients also found the combination to be safe and showed preliminary activity (with 13 of 26 patients achieving complete remission) [62]. Future studies should evaluate the role of enasidenib in combination with azacitidine and venetoclax in either the up-front or relapsed/refractory setting.

### 3.2. Ivosidenib

Ivosidenib is a selective oral inhibitor of mutant *IDH1* that received FDA approval in 2018 for relapsed/refractory *IDH1*-mutant AML and subsequently for newly diagnosed IDH1-mutant AML patients aged ≥75 years or who have comorbidities precluding intensive induction chemotherapy. Its approval was based on clinical trial data revealing an overall response rate of 54.5% in newly diagnosed patients, with a 30.3% CR rate [63]. In the relapsed/refractory setting, the overall response rate was 41.6% [64]. Similar to enasidenib, resistance to ivosidenib monotherapy is associated with RAS/RTK pathway mutations [65]. Additional resistance mechanisms have also been described, such as second-site *IDH* mutations, enhancement of “stem-ness,” upregulation of oxidative phosphorylation, and *IDH* isoform switching [66,67,68,69]. Combinatorial therapies with ivosidenib have also yielded exciting results. In the phase 3 AGILE trial of previously untreated *IDH1*-mutant AML patients unfit for intensive therapy, ivosidenib plus azacitidine significantly improved OS compared with azacitidine alone (median OS 29.3 months vs. 7.9 months with placebo-azacitidine, *p* < 0.0001) [70,71]. Importantly, the addition of azacitidine was associated with higher rates of molecular clearance in a subset of patients, as assessed by next generation sequencing. Early phase Ib/II studies combining ivosidenib with venetoclax ± azacitidine suggest promising CR rates with favorable survival outcomes [16].

### 3.3. Olutasidenib

Beyond ivosidenib and enasidenib, olutasidenib is a selective IDH1 inhibitor that received FDA approval in 2022 after demonstrating meaningful clinical activity in relapsed or refractory *IDH1*-mutated AML. In a phase 1/2 trial of R/R patients, olutasidenib achieved an overall response rate of approximately 48% with a median overall survival of 11.6 months and a median duration of response of ~26 months, including in patients previously treated with venetoclax [72]. Other early IDH1 inhibitors such as IDH305 and pan-IDH agents like BAY1436032 showed some potential antileukemic signals in early studies but were not advanced due to limited efficacy [73,74]. Dual IDH1/2 inhibitors like vorasidenib currently lack published AML-specific patient outcome data.

## 4. MENIN Targeting Agents

MENIN is a cofactor essential for leukemogenesis in *KMT2A*-rearranged (*KMT2Ar*) and nucleophosmin (*NPM1*) mutant (*NPM1m*) AML. KMT2A, also known as MLL1, is a histone H3 lysine-4 (H3K4) methyltransferase that is essential for normal hematopoiesis [75]. KMT2A interacts with MENIN, which functions as a scaffold to recruit KMT2A to chromatin, thereby promoting upregulation of *HOX* genes and their cofactor Meis homeobox 1 (*MEIS1*)—collectively referred to as the HOX/MEIS1 complex—in hematopoietic stem and progenitor cells [76,77]. This process results in a temporary state of self-renewal and proliferation, which is essential for normal hematopoiesis [78,79].

When *KMT2A* is rearranged, its N-terminus is fused with the C-terminus of a fusion partner protein, of which over 80 have been identified [80]. This fusion protein causes constitutive activation of the HOX/MEIS1 complex, resulting in differentiation blockade and leukemogenesis [81]. In vitro studies have shown that the deletion of *MEN1* or the perturbation of the *KMT2A*-MENIN interaction resulted in significantly reduced HOX/MEIS1 gene expression, suggesting that MENIN is required for activation and maintenance of *HOX* gene expression [77,82].

In *NPM1m* leukemia, the mutated NPM1 protein (NPM1c) acquires a nuclear export signal, resulting in cytoplasmic accumulation [83]. These leukemias rely on persistent *HOXA/HOXB* and *MEIS1* expression maintained by wild-type KMT2A bound to MENIN at target enhancers, resulting in aberrant *HOX* gene expression. This disease pathologically manifests similarly to a *KMT2Ar* AML, resulting in a differentiation blockade that is relieved upon blocking the MENIN-KMT2A interaction [84].

MENIN inhibitors block the KMT2A-MENIN binding site, which effectively inhibits the transcription of *HOXA* and *MEIS1*, among other leukemogenic genes [85,86]. This makes MENIN inhibitors a unique class of targeted agents that may be able to treat leukemias beyond the scope of just *KMT2Ar* and *NPM1m*, as long as the leukemia is dependent on *HOX/MEIS1* upregulation.

There are currently two MENIN inhibitors approved by the FDA to treat AML: revumenib and ziftomenib.

### 4.1. Revumenib

Revumenib (SNDX-5613-0700) was the first MENIN inhibitor approved by the FDA to treat both *KMT2Ar* and *NPM1m* R/R AML in both adults and pediatric patients 1 year of age and older. Preclinically, revumenib showed promise as a potential treatment for AML as it dramatically decreased leukemia burden in patient-derived xenograft (PDX) models of *KMT2Ar* or *NPM1m* AML [87,88]. This led to the single-arm phase I/II AUGMENT-101 trial (NCT04065399), which enrolled patients at least 30 days old with R/R *KMT2Ar* acute leukemia [89] or *NPM1m* AML [90] to receive revumenib once every 12 h, in 28-day cycles.

The *KMT2Ar* cohort met the prespecified stopping conditions for efficacy, which accelerated its FDA approval for R/R *KMT2Ar* acute leukemias in November of 2024. Specifically, the cohort exhibited a rate of complete remission (CR) plus CR with partial hematologic recovery (CRh) of 21.2% with a median CR+CRh duration of 6.4 months. For patients who achieved CR or CRh, the median time to CR or CRh was 1.9 months (range, 0.9–5.6 months). Importantly, 14% of patients who were dependent on red blood cell (RBC) and/or platelet transfusions at baseline became independent of transfusions by the 56-day post-baseline period. A total of 48% of patients who had previously been independent of RBC and platelet transfusions at baseline remained independent throughout the 56-day period.

By October 2025, revumenib was also approved to treat R/R *NPM1* AML in adult and pediatric patients 1 year and older who have no satisfactory alternative treatment options [90]. The CR+CRh rate was 23.1% (95% CI, 13.5–35.2%) with a duration of 4.5 months (95% CI, 1.2–8.1 months). A total of 17% of RBC and/or platelet transfusion-dependent patients became transfusion-independent during the 56-day post-baseline period.

Multiple phase I/II/III studies are ongoing to evaluate revumenib across additional clinical settings, including in combination regimens (most notably with venetoclax and azacitidine) and as frontline therapy (SAVE [NCT05360160]; SNDX-5613-0708 [NCT06226571]; Beat AML [NCT03013998]; EVOLVE-2 [NCT06652438]). Results from these trials will be important for defining the optimal sequencing and combinatorial use of MENIN inhibitors in *HOX*-aberrant leukemias.

### 4.2. Ziftomenib

Ziftomenib (KO-539) is another small molecule inhibitor of the MENIN-KMT2A interaction. Its structural analogue, MI-3454, induced significant regression of leukemia in *KMT2Ar* or *NPM1m* mouse models, accompanied by significantly reduced *HOXA9* and *MEIS1* levels [91]. These promising preclinical results led to the phase I/II KOMET-001 trial, which supported FDA approval of ziftomenib for the treatment of R/R *NPM1m* AML in adults who have no satisfactory alternative treatment options [92]. The CR+CRh rate was 21.4% (95% CI, 14.2–30.2%) for a duration of 5 months (95% CI, 1.9–8.1 months). A total of 21.2% of RBC and/or platelet transfusion-dependent patients became transfusion-independent during the 56-day post-baseline period, and 26.1% of transfusion-independent patients remained transfusion-independent during the 56-day period. The efficacy of ziftomenib as frontline treatment for newly diagnosed *NPM1m* and *KMT2Ar* AML is currently being tested in the ongoing phase III KOMET-017 trial (NCT07007312).

### 4.3. MENIN Inhibitor Resistance

Despite clear clinical activity of MENIN inhibitors in *KMT2Ar* and *NPM1m* acute leukemias, resistance frequently emerges with monotherapy. The best-characterized mechanism of acquired resistance is the development of on-target *MEN1* missense mutations that disrupt drug binding while preserving the MENIN-KMT2A interaction required to maintain leukemogenic HOX/MEIS1 transcriptional programs [85]. These mutations cluster within the MENIN drug-binding pocket and have been identified in patients progressing on revumenib, with strong concordance between clinically observed variants and residues enriched in CRISPR base-editing resistance screens using cell lines. Notably, ultrasensitive assays failed to detect these mutations in pretreatment samples, suggesting that resistance arises under therapeutic selection [85]. The rapid emergence of MEN1 resistance mutations underscores both the biological dependency of these leukemias on the MENIN-KMT2A axis and the limited durability of MENIN inhibitor monotherapy in the R/R setting.

In addition to genetic escape, non-genetic resistance mechanisms have been described that reflect epigenetic plasticity rather than loss of drug-target engagement. Functional genomic screens have identified altered chromatin regulation involving the KMT2C/D-UTX complex [93] and Polycomb repressive machinery [94], leading to reactivation of oncogenic transcriptional programs such as MYC despite continued MENIN inhibition. These findings suggest that some leukemias can rewire transcriptional dependencies away from canonical HOX/MEIS1 targets, enabling persistence in the presence of effective target engagement. Together, these genetic and epigenetic resistance pathways provide a strong biological rationale for combination strategies incorporating MENIN inhibitors with agents targeting complementary vulnerabilities, including BCL-2, FLT3, CDK4/6, or IKAROS degradation, with the goal of deepening responses and constraining adaptive escape mechanisms.

## 5. Conclusions

The advances made in targeted therapies for AML over the past decade have heralded a new wave of personalized oncology, wherein the treatment options for patients with targetable mutations are expanding. The application of routine genomic sequencing has made it possible for AML treatment to be personalized to each patient’s unique disease. This has been especially important in treating patients who are unfit for conventional intensive chemotherapy or with relapsed/refractory disease. However, more treatment options are needed, especially for the highest risk forms of AML, such as *TP53*-mutant or inv(3)/t(3;3), which often remain incurable even despite allogeneic stem cell transplantation. Additionally, AML remains highly prone to relapse due to clonal heterogeneity and rapid evolution under distinct selection pressures. A patient’s risk of relapse and resistance may be estimated by co-mutational burden and MRD assessment, and ongoing studies will continue to improve the detection and treatment of these cases [95,96]. Moreover, clinical trials are investigating how the sequence, combination, and/or timeline of existing targeted therapies affect outcomes for various molecular subtypes of AML. The discovery and targeting of genetic and epigenetic determinants of AML remains an exciting and promising aspect of clinical drug development and will continue to improve outcomes for AML patients.
**Drug Name****Molecular Target****FDA Approved or in Trials for AML?****Indication and Line of Therapy****Combination Regimen****Results**VenetoclaxBCL-2ApprovedNDAzacitidine, decitabineORR of 66.4% vs. 28.3% for 14.7 vs. 9.6 months [12]LisaftoclaxBCL-2In trials (phase 1b/2) R/RAzacitidineORR of 38.9% [24]SonrotoclaxBCL-2In trials (phase 1b/2)ND, R/R AzacitidinePendingNavitoclaxBCL-2 and BCL-XLIn trials (phase 1b) R/RVenetoclax and decitabineCombined CR, CRh, or CRi of 20% [26]MidostaurinFLT3-ITD and FLT3-TKDApprovedND7 + 3 (Daunorubicin cytarabine)OS of 74.7 vs. 25.6 months [43]GilteritinibFLT3-ITD and FLT3-TKDApprovedR/RN/AOS of 9.3 vs. 5.6 months [47]QuizartinibFLT3-ITDApprovedND7 + 3 (Daunorubicin cytarabine)OS of 31.9 vs. 15.1 months [54]EnasidenibIDH2ApprovedR/RN/AORR of 40.5% vs. 9.9% and EFS of 4.9 vs. 2.6 months [60]IvosidenibIDH1ApprovedND, R/RAzacitidineORR of 54.5% and CR of 30.3% (monotherapy); OS 29.3 vs. 7.9 months (combination) [63]OlutasidenibIDH1ApprovedR/RN/AORR of 48% with OS of 11.6 months [72]RevumenibMENINApprovedR/RN/ACR+CRh of 21.2% for 6.4 months in *KMT2Ar* cohort [89] CR+CRh of 23.1% for 4.5 months in *NPM1* cohort [90]ZiftomenibMENINApprovedR/RN/ACR+CRh of 21.4% for 5 months [92]ND = newly diagnosed; R/R = relapsed/refractory; ORR = overall response rate; CR = complete remission; CRh = complete remission with partial hematologic recovery; CRi = complete remission with incomplete hematologic recovery; EFS = event-free survival; N/A = not applicable.

## Data Availability

No new data were created or analyzed in this study. Data sharing is not applicable to this article.
